# Depression and social phobia in essential tremor and Parkinson's disease

**DOI:** 10.1002/brb3.781

**Published:** 2017-08-02

**Authors:** Ligita Smeltere, Vladimirs Kuzņecovs, Renārs Erts

**Affiliations:** ^1^ Health Center 4 Consulting Room for Parkinson's Disease and Other Movement Disorders Riga Latvia; ^2^ Medical Faculty of University of Latvia Riga Latvia; ^3^ Riga Center of Psychiatry and Addiction Disorders Riga Latvia

**Keywords:** depression, essential tremor, Parkinson's disease, social phobia

## Abstract

**Background:**

Essential tremor (ET) and Parkinson's disease (PD) are the two most common movement disorders, and tremor is the most visible symptom. Comparative study on ET and PD clinical neuropsychiatric symptoms was performed to assess the impact of emotional state on tremor.

**Objectives:**

To investigate the most common psychiatric symptoms (depression, anxiety and social phobia) and their correlations with motor symptoms, especially tremor, in ET and PD patients.

**Materials and Methods:**

This comparative cross‐sectional study consisted of neurological examinations, five self‐assessment questionnaires (Depression Anxiety Stress Scale [DASS], Beck Depression Inventory [BDI], Social Interaction Anxiety Scale [SIAS], Social Phobia Scale [SPS], and State‐Trait Anxiety Inventory[STAI]), clinical interviews with 45 ET patients, 40 PD patients, and 40 controls (CG), and statistical analysis was performed for 40:35:39 respectively.

**Results:**

BDI revealed depressive disorders of various severities in all groups (ET=79.5%, PD=91.2%, and CG=66.7%). The study found no significant difference between ET and PD groups (*p* = .708) and significant difference between the patients and controls (ET/CG *p* = .049; PD/CG *p* = .007).

Depression (DASS(D), BDI) did not correlate with tremor severity (*p* > .05) in ET and PD patients.

The prevalence of social phobia was ET=50.0%, PD=42.9%, and CG=20.5%. There was significant difference between ET/CG (SIAS *p* = .02, SPS *p* = .001) and PD/CG (SPS *p* = .018), but no difference between ET and PD groups (*p* > .05). Tremor and SPS moderately correlated in ET patients (*r* = .35, *p* = .02).

**Conclusions:**

ET and PD patients showed high comorbidity of psychiatric disorders, but there was no significant difference between these two groups. ET severity correlated with social phobia scale scores.

## 1 INTRODUCTION

The aim of this study was to investigate neurological and psychiatric symptoms in Latvian patients with the two most common (Fahn, Jankovic, & Halett, 2011) movement disorders: essential tremor (ET) and Parkinson's disease (PD). We assessed the severity, characteristics, and prevalence of each symptom, and explored the links between motor symptoms, especially tremor, and mental disorders.

Although ET was initially believed to be a monosymptomatic disease, it is now considered a polysymptomatic disease with motor (tremor, gait ataxia, and postural instability) and nonmotor (cognitive, psychiatric, and sensory) (Louis, 2010; Teive, 2012) symptoms.

A lot of studies have been conducted to investigate nonmotor symptoms, especially depression, in PD patients (Reijnders, Ehrt, Lousberg, Aarsland, & Leentjens, 2009; Riedel et al., 2010; Schrag, Hovris, Morley, Quinn, & Jahanshahi, 2003) and to identify its traits, including the disease appearance at the premotor stage, the severity variability, and the prevalence of apathy, anhedonia, hopelessness, and lack of energy in the symptomatology.

ET is characterized by variable tremor frequency and amplitude fluctuations up to 50%, despite standardization of recordings and repeated accelerometer measurements (Bain, 2005; Cleeves & Findley, 1987). Many other factors also affect ET, including the physical and mental condition of the patient (e.g., emotional state), natural tremor changes, and other factors. Personal clinical experience led us to focus on the presence of anxiety, cognitive and behavioral traits, and their effects on tremor in ET and PD patients.

In Latvia, patients with movement disorders are mostly consulted and treated by a neurologist in cooperation with a GP. Only a small number of patients turn for help at specialized movement disorder consultations. The analyzed anamnesis data and medical records revealed that quite often ET is poorly recognized and/or misdiagnosed as PD (30% erroneous ET diagnoses) in Latvia (Smeltere & Smeltere, 2015); other authors (Louis & Ferreira, 2010; Romero, Benito‐Leon, & Bermejo‐Pareja, 2012) have also reported this problem. Moreover, psychiatric disorders are rarely acknowledged because of the stigma of visiting a psychiatrist.

When starting the present study, the literature did not offer comparative investigations between ET and PD clinical neuropsychiatric symptoms. Thus, Latvian population sample was selected to research the symptoms associated with these diseases. This study was performed by the author of the present article L. Smeltere, who specializes in movement disorders. The study consisted of a detailed neurological examination, diagnostic tests for depression and anxiety, especially social anxiety (phobia), and an assessment of personality traits related to anxiety. A hypothesis was proposed that emotional states can cause changes in tremor severity and vice versa. Recognizing the mental traits or disorders on the psychiatric spectrum might result in changes in future treatment tactics and improve the quality of life and well‐being of patients.

## 2 METHODS

### 2.1 Participants

ET and PD patients were recruited from the Neurology Outpatient Center at Pauls Stradiņš Clinical University Hospital and the Consulting Room for Parkinson's Disease and Other Movement Disorders at Health Center 4 during the period September 2013–January 2015. Patients came from different regions of Latvia. All patients who came for a consultation and had definite ET or PD diagnosis were asked to participate in the study; thus the selection was made.

The study enrolled 45 ET patients, 40 PD patients, and 40 controls (hereafter CG), all of whom provided signed written informed consent. Eleven patients had to withdraw from the study because they only partially completed the forms for various reasons (e.g., lack of time, business reasons, motor disturbances, and tiredness). The data of included 40 ET and 35 PD patients, and 39 controls were used for statistical analysis. CG consisted of healthy, age‐ and gender‐matched individuals without ET and PD. However, while the elderly individuals displayed different somatic disorders, including arterial hypertension, adiposity, and osteoarthritis (reflecting the real life situation of seniors), we excluded patients with decompensated somatic disorders. No other exclusion criteria were established. The research was approved by the hospital and Ethics Committee of the University of Latvia.

### 2.2 Study evaluation

Only patients with a definite ET or PD diagnosis (confirmed by L.S.) were included. ET was diagnosed on the basis of anamnesis data for tremor lasting at least 5 years, the criteria of the Tremor Investigation Group (TRIG) (Deuschl, Bain, & Brin, 1998), a clinical assessment of tremor according to the Fahn, Tolosa, Marin tremor rating scale (TRS) (Fahn, Tolosa, & Marin, 1988), and the MDS diagnostic inclusion and exclusion criteria for ET (Deuschl et al., 1998). PD was diagnosed on the basis of a neurological examination, including the UPDRS (Fahn & Elton, 1987) Part III (motor) (the lead author is a certified MDS PD rater) and the diagnostic criteria for PD (UK PDS Brain Bank's Criteria for Idiopathic Parkinson's Disease) (Berardelli et al., 2013; Hughes, Daniel, Kilford, & Lees, 1992). The PD patients fell between stages I and IV on the Hoehn & Yahr (H&Y) scale. The Montreal cognitive assessment (Nazem et al., 2009) was used to test stage IV patients, excluding patients with cognitive dysfunctions. The cut‐off score was <24. Patients with stage V PD were not included because all patients in this stage display pronounced cognitive dysfunction. Brain MRI and a clinical analysis were performed for both patient groups to exclude other phenotypically similar diseases. Patients with comorbid ET‐PD diseases were not included.

Within the framework of this cross‐sectional study, all tests were conducted once. The neurological condition of the patients was assessed, including Fahn, Tolosa, Marin TRS for ET and UPDRS for PD patients. All participants were asked to complete the following five internationally recognized, reliable, valid and consistent self‐assessment forms in their native language: 1) DASS—Depression Anxiety Stress scale (Lovibond & Lovibond, 1995), which diagnoses the locus and degree of emotional disturbance (higher scores = more severe), 2) BDI‐II—Beck Depression Inventory—second edition (Beck, Steer, & Brown, 1996), where scores ranging from 6–13 indicate minimal depression, 14–19 indicate mild depression, 20–28 indicate moderate depression, and 29–63 indicate severe depression; 3) SIAS—Social Interaction Anxiety Scale (to assess distress when meeting and talking with other people) (Mattick & Clarke, 1998), 4) SPS—Social Phobia Scale (to assess fears of being scrutinized during routine activities, e.g., eating, drinking, and writing) (Mattick & Clarke, 1998), and 5) the STAI test–State‐Trait Anxiety Inventory form Y (Spielberger, Gorush, & Lushene, 1970). For SIAS and SPS, items are rated from 0 (not characteristic or true of me) to 4 (extremely characteristic), and both scales are scored by summing up all items, with higher scores indicating a more impaired state. STAI was used to differentiate between anxiety as a condition (State anxiety Y‐1) and anxiety as a personality trait, meaning a predisposition to experience persistent anxious behavior (Trait anxiety Y‐2). Y‐1 was used for all participants to evaluate how they felt at a given moment and to assess state anxiety caused by the disease, whereas Y‐2 was used to determine how they felt in general. STAI items are scored on a 4‐point scale with a total score range of 20–80 for each scale. A score between 20 and 39 indicates low anxiety, 40–59 indicates moderate anxiety, and 60–80 indicates high anxiety. The total score indicates the prevailing type of anxiety. Trait factors predict better person's behavior than state factors, although under extreme situations, state factors can have a crucial effect on an individual's behavior. The level of trait anxiety indicates the disposition of an individual to perceive an objectively safe situation as threatening and dangerous and to respond to it with a state of anxiety, the intensity of which is not objectively appropriate for the situation. The self‐assessment results were complemented by performing clinical interviews to ensure objectivity and to diagnose any possible comorbidities according to ICD‐10 (International Classification of Diseases ICD‐10, 2015) and DSM‐5 (American Psychiatric Association, 2013) classifications.

### 2.3 Statistical analysis

Statistical analysis was performed using the SPSS program (IBM SPSS Statistics Version 22, SPSS Inc., USA). A *p <* .05 was considered to indicate statistical significance.

Demographic and clinical characteristics were compared across three groups using analysis of variance (ANOVA), Pearson's chi‐squared tests and Kruskal–Wallis tests. Eta squared (ɳ^2^) was used to calculate the effect size where 0.01 was accepted as small, 0.06—moderate, 0.13—large. Cut‐off scores were not used.

We hypothesized that there would be a lower rate of depression in ET than in the PD patients according to the results of the BDI scale and social phobia scores would be higher in ET patients than in PD patients. Next, we compared each disease group with other groups (ET/PD, ET/CG, PD/CG) to determine differences using post hoc analysis with a Tukey correction in all of the self‐assessment tests.

The mean of every BDI symptom and scale (SIAS and SPS) statement results were calculated to compare them across all study groups.

We searched for tremor correlations with psychic symptoms because tremor is a sign similar in both (ET and PD) diseases. To test the hypothesis that emotional states cause changes in tremor severity and vice versa, we compared the results of the Fahn, Tolosa, Marin TRS, UPDRS (all motor scores and separately for tremor) with DASS, BDI, SIAS, and SPS tests using Pearson's r or Spearman's r_s_ correlation coefficient.

Cronbach's alpha was used to measure internal consistency in the SIAS and SPS tests.

## 3 RESULTS

A total of 114 participants completed the study: 40 ET, 39 PD, and 35 CG.

Demographic factors and clinical characteristics are summarized in Table 1.

**Table 1:** Demographic and clinical characteristics of the study groups

	ET	PD	CG	*p* value
Number (n)	40	35	39	
Age, Mean (±SD)	52.05 (20.18)	61.51 (9.02)	55.85 (16.36)	.094^a^
Female, (%)	26 (65%)	19 (54%)	29 (74%)	.34^b^
Fahn, Tolosa, Marin tremor rating scale mean (±SD)	28.98 (20.14)			
UPDRS III Mean (±SD)		25.49 (13.44)		
DASS				
Mean (±SD)	35.63 (22.32)	33.29 (19.21)	21.33 (17.70)	.004^c^
Difference *p* value^d^	ET/CG .005^d^	PD/CG .03^d^		
Difference *p* value^d^	ET/PD .867^d^			
BDI				
Mean,(±SD)	14.87 (11.56)	16.37 (9.28)	9.64 (7.82)	.008^c^
Difference *p* value^d^	ET/CG .049^d^	PD/CG .007^d^		
Difference *p* value^d^	ET/PD .708^d^			
SIAS				
Mean (±SD)	21.68 (14.96)	17.49 (11.06)	14.18 (9.88)	.027^c^
Difference *p* value^d^	ET/CG .02^d^	PD/CG .479^d^		
Difference *p* value^d^	ET/PD .304^d^			
SPS				
Mean (±SD)	20.80 (17.66)	18.11 (15.19)	8.85 (.82)	.001^c^
Difference *p* value^d^	ET/CG .001^d^	PD/CG .018		
Difference *p* value^d^	ET/PD .70^d^			
STAI				
State anxiety Y‐1, Mean, (±SD)	45.43 (13.87)	44.57 (11.88)	37.03 (10.14)	.005^c^
Difference *p* value^d^	ET/CG .007^d^	PD/CG .023^d^		
Difference *p* value^d^	ET/PD .95^d^			
Trait anxiety Y‐2, Mean, (±SD)	41.08 (8.92)	37.94 (7.2)	42.0 (9.7)	.119^c^

ET, essential tremor; PD, Parkinson's disease; CG, control group; SD, standard deviation; UPDRS, Unified Parkinson's Disease Rating Scale, part III; DASS, Depression Anxiety Stress scale; BDI, Beck Depression Inventory; SIAS, Social Interaction Anxiety Scale; SPS, Social Phobia Scale; STAI, State‐Trait Anxiety Inventory, form Y.

Values are shown as the mean (±SD, standard deviation) or number (percentage).

a Kruskal–Wallis test.

b Pearson's chi‐squared test.

c Analysis of variance (ANOVA).

d Post hoc analysis with Tukey correction.

The primary results showed that there were statistically significant differences in study groups in all tests: DASS (*F* (2, 111) = 5.78; *p* = .004, ɳ^2^=.17), BDI (*F* (2, 109) = 5.30; *p* = .006, ɳ^2^=.09, ANOVA), SIAS (*F* (2,111)=3.72; *p* = .027; ɳ^2^ =.06), and SPS (*F* (2,111)=7.42; *p* = .001; ɳ^2^=.13). However, further analysis showed that the mean scores of all tests were not statistically different between ET group and PD group (*p* > .05), whereas there was a significant difference between ET and CG groups (*p* < .05), and between PD and CG groups (*p* < .05). To better recognize symptoms of depression, BDI tests were performed on the same day. DASS (D) and BDI test results moderately or closely correlated (*p* < .001) in all groups.

BDI revealed the presence of symptoms with different depression severity levels in 79.49% of the ET patients, 91.18% of the PD patients, and 66.67% of CG. These results were confirmed during the clinical interview. Although in practice, depression seemed to be more severe in PD than in ET patients, our data did not show statistical significance (*p* > .708).

**Figure 1:**
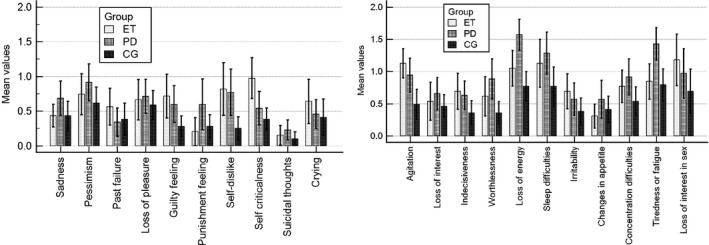
Mean values of Beck Depression Inventory (BDI). Figure shows means and standard deviations of 21 symptoms of depression and the comparison of three groups. Mean values were calculated from patient's answers, where items are scored on a 4‐point scale (0–3 scale) according to increasing severity

When we compared the means of every symptom of depression (BDI), we found that ET patients displayed some differences. The following were the most prominent: loss of interest in sex^a^ (*M* = 1.18), agitation^b^ (*M* = 1.13) and self‐criticalness^c^ (*M* = .97) (respectively, for PD: *M* = .97^a^, .94^b^, and .54^c^). PD patients displayed a loss of energy^d^ (*M* = 1.57), tiredness or fatigue^e^ (*M* = 1.43) and changes in sleeping patterns^f^ (*M* = 1.29) vs. ET (*M* = 1.05^d^, 1.18^e^, and 1.13^f^). It is interesting that CG showed the same symptoms of depression as the PD and ET patients but with a lower intensity (see Figure 1).

In SIAS and SPS tests, all the groups showed good internal consistency (Cronbach's α > 0.9).

A comparison of the mean SIAS (Smeltere, Kuznecovs, & Smelters, 2015) scores revealed that ET patients were more worried that they would not know what to say in social situations (*M* = 1.54) than PD patients (*M* = 1.17) or CG (*M* = 1.03) were. ET patients were more nervous when asked to speak with someone in authority (*M* = 1.51) than PD patients (*M* = 1.03). PD patients felt more anxious when mixing socially (M_PD_=1.37; M_ET_=1.33) and when speaking about themselves or their feelings^g^ (*M* = 1.34) or expressing themselves^h^ (*M* = 1.26), but the mean values for these characteristics were higher in the ET group (*M* = 1.49^g^; 1.44^h^). The CG had a mix of the same symptoms but showed lower scores.

**Figure 2:**
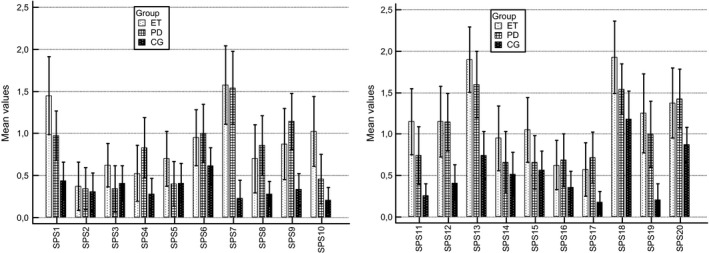
Mean values of Social Phobia Scale (SPS). Statements of SPS—1: I become anxious if I have to write in front of other people; 2: I become self‐conscious when using public toilets; 3: I can suddenly become aware of my own voice of others listening to me; 4: I get nervous that people are staring at me as I walk down the street; 5: I fear I may blush when I am with others; 6: I feel self‐conscious if I have to enter a room where others are already seated; 7: I worry about shaking or trembling when I'm watched by other people; 8: I would get tense if I had to sit facing other people on a bus or a train; 9: I get panickly that others might see me faint or be sick or ill; 10: I would find it difficult to drink something if in a group of people; 11: It would make me feel self‐conscious to eat in front of a stranger at a restaurant; 12: I am worried people will think my behavior odd; 13: I would get tense if I had to carry a tray across a crowded cafeteria; 14: I worry I'll lose control of myself in front of other people; 15: I worry I might do something to attract the attention of other people; 16: When in an elevator, I am tense if people look at me; 17: I can feel conscious standing in a line; 18: I can get tense when I speak in front of other people; 19: I worry my head will shake or nod in front of others; 20: I feel awkward and tense if I know people are watching me. The comparison of social phobia symptomes (statements) in ET, PD, and control groups. Mean values were calculated from patient's answers, where: 0 =  not at all characteristic or true of me, 1 =  slightly characteristic or true of me, 2 =  moderately characteristic or true of me, 3 =  very characteristic or true of me, 4 =  extremely characteristic or true of me

SPS tests (see Figure 2) showed that speaking in front of other people caused the highest tension in ET patients (*M* = 1.93), followed by PD patients (*M* = 1.54) and CG (*M* = 1.18). Carrying a tray across a crowded cafeteria was the second most stressful situation for both ET and PD patients. This situation was more stressful for the ET (*M* = 1.9) than for the PD patients (*M* = 1.6) and caused less stress in the CG (*M* = .74). The statement, “I worry about shaking or trembling when I'm watched by other people,” was characteristic of both the ET (*M* = 1.58) and PD (*M* = 1.54) groups.

Taking into account SIAS, SPS, other tests and the clinical interview, social phobia was clinically diagnosed in 20 ET patients (50%), 15 PD patients (42.86%), and 8 individuals in the CG (20.51%).

In assessing the effect of depression on tremor, we found that ET tremor severity and PD rest tremor were not correlated with BDI scores or with DASS depression, anxiety, or stress subscales scores (*p* > .05).

When estimating the influence of social anxiety on tremor, we found a moderate but statistically significant correlation between ET severity and SPS scores (r_s_= .35; *p* = .02). PD group showed an average correlation between the stage of the disease and SPS scores (r = .47; *p* = .004).

The STAI State anxiety (Y‐1) results showed that there was a significant difference between ET/CG and PD/CG (*p* < .05) but no difference between ET/PD. Trait anxiety (Y‐2) was not different between the groups (*p* = .119). Different emotional reactions to stressful situations could be predicted by analyzing Y‐1 and Y‐2. The following reactions were the most common in ET patients: 32.5% had low‐level anxiety both as a personality trait and as a reaction to the disease at the time they completed the form, 20% experienced a moderate level of anxiety both as a personality trait and as a reaction to the situation (in these cases, it was difficult for the patient to calm down and adapt to the situation, and the clinical interview confirmed the diagnosis of social anxiety disease in all of these patients), 20% had a moderate level of anxiety as a personality trait but high‐level anxiety because of the disease. The state anxiety that resulted from the disease prevailed/dominated over the personality trait in 37.5% of the ET patients. In PD group, 88.5% manifested low or moderate anxiety both as a personality trait and as a reaction to the situation.

## 4 DISCUSSION

Two different self‐assessment tests for diagnosing depression were used to identify depressive conditions. However, the results differed between the two tests that were performed on the same day; the BDI test was more sensitive. The tests showed that there was no statistically significant difference between ET and PD, likely as a result of the small sample size. Using SIAS and SPS allowed us to diagnose social phobia which had a similar prevalence in ET and PD patient groups. SIAS and SPS identify situations in which both ET and PD patients tended to exhibit symptoms. This finding should be taken into account when practicing cognitive behavioral therapy (CBT) for patients with motor disturbances. The fact that ET severity was correlated with SPS supports the hypothesis that emotional states cause changes in tremor severity or vice versa but does not indicate which is primary (therefore further research is needed). These findings allow us to propose a new hypothesis that social phobia might play role in the tremorogenesis process in ET patients (as tremor amplitude‐enhancing factor).

Based on the STAI test results, we conclude that the lower the anxiety level (as a symptom and a personality trait), the likelihood of developing social anxiety (phobia) decreases and vice versa. Moderate anxiety was a personality trait in 49% of the controls, indicating that anxiety cannot be clearly defined as a specific personality trait of ET patients.

The literature reports that the prevalence of depression is 25–54% (Tandberg, Larsen, Aarsland, & Cummings, 1996; Fabbrini et al., 2012; Louis, Benito‐León, & Bermejo‐Pareja, 2007; Reijnders, Ehrt, Weber, Aarsland, & Leentjens, 2008) in ET and PD patients, but the prevalence was noticeably higher in our sample of Latvian individuals. In our research, approximately 59% of the patients were diagnosed with minimal or mild depression, and only 17.6% met the criteria of major depression; the latter value is similar to that pointed out in a meta‐analysis (Reijnders et al., 2008) of PD depressive disorders. This prevalence is also similar in the prevalence in ET patients, of which 15% were diagnosed with major depression. These results indicate a very high prevalence of nonmotor psychiatric symptoms. Previous studies identified only moderate and severe depression in PD patients. The results of the current self‐assessment tests allow us to diagnose depression better and recognize its phenotypic traits in Latvian population. When comparing our sample to NEDICES data, in which depression was self‐reported in 43.8% of ET patients and 26% of the CG (Romero et al., 2012; Louis et al., 2007), we found that our study showed higher values in all groups. It is likely that our population's psyche has been affected by historical conditions and older adults may have experienced difficulties in adapting to the new sociopolitical conditions. Comorbid diseases and socioeconomic situations, a deficit of sunlight, and weather conditions may also have affected these patients.

Movement disorder clinics have reported that social anxiety in these patients falls within the range of 33–42% (Lundervold, Ament, & Holt, 2013; Schneier, Barnes, Albert, & Louis, 2001; Topçuoğlu et al., 2006). Our data and clinical experiences indicate that a mild manifestation of this disorder also increases the amplitude of patient's tremor in social situations. The CG indices largely coincided with those that were reported by H.M. Tharwani (Tharwani & Davidson, 2001).

Latvian patients often complain about somatic disorders, mentioning an increase in tremor when they are anxious as a subjective minor factor. It might be social and cultural differences in the Latvian population to hide inner experiences rather than express them. Social anxiety diseases are characterized by fear or anxiety about one or more social situations in which the individual is exposed to the possible scrutiny of others (American Psychiatric Association, 2013). Patients avoid speaking about how much the reactions of others worry them, including the possibility of being subjected to scrutiny, about anything that is said about them, or about the negative expectation or fear that it might occur.

L.M. Shulman et al. (Shulman, Taback, Rabinstein, & Weiner, 2002) have observed that depression and other nonmotor symptoms are poorly recognized in PD patients during routine visits. Our study showed similar data. The visual appearance of movement disorder patients might be the cause of wrong conclusions about psychiatric disturbances without conducting special tests. The test results changed our previous conclusions, which were based on clinical experience, regarding affective disturbances in ET and PD patients. Thus, practitioners should consider changing the traditional neurological approach to neuropsychiatric for patients with movement disorders. There is a need to develop a clinically proven algorithm for diagnosing and treating nonmotor symptoms in ET and PD patients. We believe that there is a necessity for further clinical trials to identify an effective pharmacotherapy for social anxiety disorder and depression in patients with tremor.

## FINANCIAL DISCLOSURES

None (all authors).

## CONFLICT OF INTEREST

None (all authors).

## AUTHORS’ CONTRIBUTIONs

LS: Conceptualized and organized the research project, executed (obtaining data) and statistical analyzed the data, and prepared and wrote the manuscript; VK (co‐author): Provided recommendations to the project, critically reviewed the research processes, and prepared the manuscript; RE (co‐author): Studied the methodology, statistical analyzed the date, and prepared the manuscript.

## References

[brb3781-bib-0001] American Psychiatric Association (2013). Diagnostic and statistical manual of mental disorders. 5th Ed. DSM‐5. www.appi.org/pages/dsm.aspx.

[brb3781-bib-0002] Bain, P . (2005). The clinical assessment of essential tremor In Lyons K. E. & Pahwa R. (Eds.), Handbook of essential tremor and other tremor disorders (p. 94). Boca Raton, FL: CRC Press https://doi.org/10.1201/b14115-9

[brb3781-bib-0003] Beck, A. T. , Steer, R. A. , & Brown, G. K. (1996). Manual for the BDI‐II. San Antonio, TX: The Psychological Corporation.

[brb3781-bib-0004] Berardelli, A. , Wenning, G. K. , Antonini, A. , Berg, D. , Bloem, B. R. , Bonifati, V. , … Vidailhet, M. (2013). EFNS/MDS‐ES/ENS [corrected] recommendations for the diagnosis of Parkinson's disease. European Journal of Neurology, 20, 16–34. https://doi.org/10.1111/ene.12022 23279440 10.1111/ene.12022

[brb3781-bib-0005] Cleeves, L. , & Findley, L. J. (1987). Variability in amplitude of untreated essential tremor. Journal of Neurology, Neurosurgery and Psychiatry, 50, 704–708. https://doi.org/10.1136/jnnp.50.6.704 10.1136/jnnp.50.6.704 PMC1032074 3612150

[brb3781-bib-0006] Deuschl, G. , Bain, P. , & Brin, M. (1998). Consensus statement of the Movement Disorder Society on tremor (diagnostic criteria for essential tremor). Movement Disorders, 13(Suppl 3), 2–23. https://doi.org/10.1002/mds.870131303 10.1002/mds.870131303 9827589

[brb3781-bib-0007] Fabbrini, G. , Berardelli, I. , Falla, M. , Moretti, G. , Pasquini, M. , Altieri, M. , … Berardelli, A. (2012). Psychiatric disorders in patients with essential tremor. Parkinsonism & Related Disorders, 18, 971–973. https://doi.org/10.1016/j.parkreldis.2012.05.005 22658234 10.1016/j.parkreldis.2012.05.005

[brb3781-bib-0008] Fahn, S. , & Elton, R. L. (1987). Members of the UPDRS Development Committee. Unified Parkinson's disease rating scale In Fahn S., Marsden C. D., Calne D. B. & Lieberman A. (Eds). Recent developments in Parkinson's disease. Vol. II (p 153‐163). Florham Park, NJ: Macmillan Health Care Information.

[brb3781-bib-0009] Fahn, S. , Jankovic, J. , & Hallett, M. (2011). Principles and practice of movement disorders. Saunders, Elsevier; p 5.

[brb3781-bib-0010] Fahn, S. , Tolosa, E. , & Marin, C. (1988). Clinical rating scale for tremor In Jankovic J., & Tolosa E. (Eds.), Parkinson's disease and movement disorders (pp. 225–234). Baltimore: Urban & Schwarzenberg.

[brb3781-bib-0011] Hughes, A. J. , Daniel, S. E. , Kilford, L. , & Lees, A. J. (1992). Accuracy of clinical diagnosis of idiopathic Parkinson's disease: A clinico‐pathological study of 100 cases. Journal of Neurology, Neurosurgery and Psychiatry, 55, 181–184. https://doi.org/10.1136/jnnp.55.3.181 10.1136/jnnp.55.3.181 PMC1014720 1564476

[brb3781-bib-0012] International Classification of Diseases ICD‐10 2015 www.who.int/classifications/icd/icdonlineversions/en/.

[brb3781-bib-0013] Louis, E. D. (2010). Essential tremor as a neuropsychiatric disorder. Journal of the Neurological Sciences, 289, 144–148. https://doi.org/10.1016/j.jns.2009.08.029 19720384 10.1016/j.jns.2009.08.029 PMC2813410

[brb3781-bib-0014] Louis, E. D. , Benito‐León, J. , & Bermejo‐Pareja, F. (2007). Neurological Disorders in Central Spain (NEDICES) Study Group. Self‐reported depression and anti‐depressant medication use in essential tremor: Cross‐sectional and prospective analyses in a population‐based study. European Journal of Neurology, 14, 1138–1146. https://doi.org/10.1111/j.1468-1331.2007.01923.x 17708753 10.1111/j.1468-1331.2007.01923.x

[brb3781-bib-0015] Louis, E. D. , & Ferreira, J. J. (2010). How common is the most common adult movement disorder? Update on the worldwide prevalence of essential tremor. Movement Disorders, 25, 534–541. https://doi.org/10.1002/mds.22838 20175185 10.1002/mds.22838

[brb3781-bib-0016] Lovibond, S. H. , & Lovibond, P. F. (1995). Manual for the depression anxiety stress scales. Sydney: NSW, the Psychology Foundation of Australia.

[brb3781-bib-0017] Lundervold, D. A. , Ament, P. A. , & Holt, P. (2013). Social anxiety, tremor severity, and tremor disability: A search for clinically relevant measures. Psychiatry, 2013, 257459 https://doi.org/10.1155/2013/257459 10.1155/2013/257459 PMC3820092 24236276

[brb3781-bib-0018] Mattick, R. P. , & Clarke, J. C. (1998). Development and validation of measures of social phobia scrutiny fear and social interaction anxiety. Behavior Research and Therapy, 36, 455–470. https://doi.org/10.1016/S0005-7967(97)10031-6 10.1016/s0005-7967(97)10031-6 9670605

[brb3781-bib-0019] Nazem, S. , Siderowf, A. D. , Duda, J. E. , Have, T. T. , Colcher, A. , Horn, S. S. , … Weintraub, D. (2009). Montreal cognitive assessment performance in patients with Parkinson's disease with “normal” global cognition according to Mini‐mental state examination score. Journal of the American Geriatrics Society, 57, 304–308. https://doi.org/10.1111/j.1532-5415.2008.02096.x 19170786 10.1111/j.1532-5415.2008.02096.x PMC2754699

[brb3781-bib-0020] Reijnders, J. S. , Ehrt, U. , Lousberg, R. , Aarsland, D. , & Leentjens, A. F. (2009). The association between motor subtypes and psychopathology in Parkinson's disease. Parkinsonism & Related Disorders, 15, 379–382. https://doi.org/10.1016/j.parkreldis.2008.09.003 18977165 10.1016/j.parkreldis.2008.09.003

[brb3781-bib-0021] Reijnders, J. S. , Ehrt, U. , Weber, W. E. , Aarsland, D. , & Leentjens, A. F. (2008). A systematic review of prevalence studies of depression in Parkinson's disease. Movement Disorders, 23, 183–189. [Quiz:313]https://doi.org/10.1002/mds.21803 17987654 10.1002/mds.21803

[brb3781-bib-0022] Riedel, O. , Klotsche, J. , Spottke, A. , Deuschl, G , Förstl, H. , Henn, F. , … Wittchen, H. U. (2010). Frequency of dementia, depression, and other neuropsychiatric symptoms in 1,449 outpatients with Parkinson's disease. Journal of Neurology, 257, 1073–1082. https://doi.org/10.1007/s00415-010-5465-z 20140443 10.1007/s00415-010-5465-z

[brb3781-bib-0023] Romero, J. P. , Benito‐Leon, J. , & Bermejo‐Pareja, F. (2012). The NEDICES Study: Recent advances in the understanding of the epidemiology of essential tremor. Tremor and Other Hyperkinetic Movements (NY), 2, PMCID: PMC3570054 DOI: 10.7916/D8N58K4H. pii: tre‐02‐70‐346‐2. 10.7916/D8N58K4H PMC3570054 23439396

[brb3781-bib-0024] Schneier, F. R. , Barnes, L. F. , Albert, S. M. , & Louis, E. D. (2001). Characteristics of social phobia among persons with essential tremor. Journal of Clinical Psychiatry, 62, 367–372. https://doi.org/10.4088/JCP.v62n0511 11411820 10.4088/jcp.v62n0511

[brb3781-bib-0025] Schrag, A. , Hovris, A. , Morley, D. , Quinn, N. , & Jahanshahi, M. (2003). Young‐versus older‐onset Parkinson's disease: Impact of disease and psychosocial consequences. Movement Disorders, 18, 1250–1256. https://doi.org/10.1002/mds.10527 14639664 10.1002/mds.10527

[brb3781-bib-0026] Shulman, L. M. , Taback, R. L. , Rabinstein, A. A. , & Weiner, W. J. (2002). Non‐recognition of depression and other non‐motor symptoms in Parkinson's disease. Parkinsonism & Related Disorders, 8, 193–197. https://doi.org/10.1016/S1353-8020(01)00015-3 12039431 10.1016/s1353-8020(01)00015-3

[brb3781-bib-0027] Smeltere, L. , Kuznecovs, V. , & Smelters, R. (2015). Research on social anxiety in patients with essential tremor and Parkinson's disease in a sample of the Latvian population. Proceedings of the Latvian Academy of Sciences Section B, 69, 250–258.

[brb3781-bib-0028] Smeltere, L. , & Smeltere, E. (2015). Research on characteristics of essential tremor in the Latvian population. Proceedings of the Latvian Academy of Sciences Section B, 69, 259–264.

[brb3781-bib-0029] Spielberger, C. D. , Gorush, R. L. , & Lushene, R. E. (1970). Manual for the state‐trait anxiety inventory. Palo Alto, Calif.: Consulting Psychologists Press.

[brb3781-bib-0128] Tandberg, E. , Larsen, J. P. , Aarsland, D. , & Cummings, J. L. (1996). The occurrence of depression in Parkinson’s disease. A community‐based study. Archives of Neurology, 53, 175–179. https://doi.org/10.1001/archneur.1996.00550020087019. 8639068 10.1001/archneur.1996.00550020087019

[brb3781-bib-0030] Teive, H. A. (2012). Essential tremor: Phenotypes. Parkinsonism & Related Disorders, 18(Suppl 1), S140–S142. https://doi.org/10.1016/S1353-8020(11)70044-X 22166415 10.1016/S1353-8020(11)70044-X

[brb3781-bib-0031] Tharwani, H. M. , & Davidson, J. R. (2001). Symptomatic and functional assessment of social anxiety disorder in adults. Psychiatric Clinics of North America, 24, 643–659. https://doi.org/10.1016/S0193-953X(05)70255-0 11723625 10.1016/s0193-953x(05)70255-0

[brb3781-bib-0032] Topçuoğlu, V. , Bez, Y. , Sahin Biçer, D. , Dib, H. , Kuşçu, M. K. , Yazgan, C. , … Göktepe, E. (2006). Social phobia in essential tremor. Turk Psikiyatri Derg, 17, 93–100. 16755409

